# Predicting Chronological Age via the Skin Volatile Profile

**DOI:** 10.1021/jasms.3c00315

**Published:** 2024-02-07

**Authors:** Melissa Finnegan, Shane Fitzgerald, Romain Duroux, Joan Attia, Emma Markey, David O’Connor, Aoife Morrin

**Affiliations:** † School of Chemical Sciences, Insight SFI Research Centre for Data Analytics, National Centre for Sensor Research, Dublin City University, Dublin D09 V209, Ireland; ‡ IFF-Lucas Meyer Cosmetics, Toulouse, Cedex 1, 31036, France

## Abstract

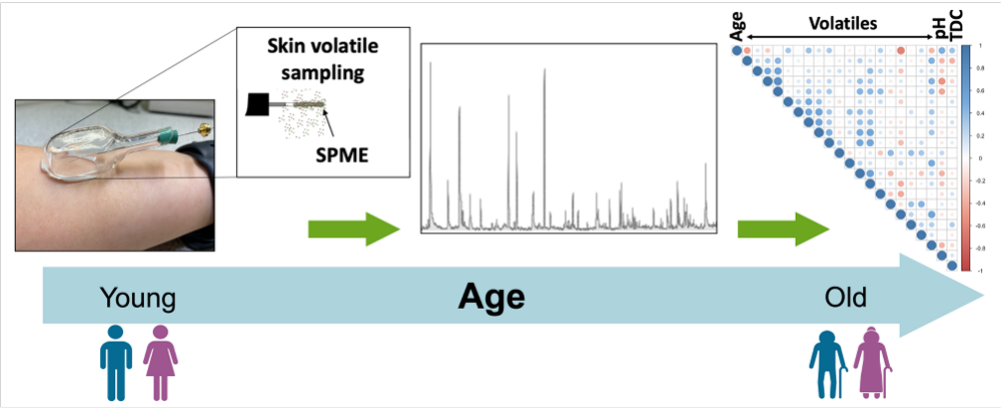

Skin volatile emissions offer a noninvasive insight into metabolic activity within the body as well as the skin microbiome and specific volatile compounds have been shown to correlate with age, albeit only in a few small studies. Building on this, here skin volatiles were collected and analyzed in a healthy participant study (*n* = 60) using a robust headspace-solid phase microextraction (HS-SPME) gas chromatography–mass spectrometry (GC-MS) workflow. Following processing, 18 identified compounds were deemed suitable for this study. These were classified according to gender influences and their correlations with age were investigated. Finally, 6 volatiles (of both endogenous and exogenous origin) were identified as significantly changing in abundance with participant age (*p* < 0.1). The potential origins of these dysregulations are discussed. Multiple linear regression (MLR) analysis was employed to model age based on these significant volatiles as independent variables, along with gender. Our analysis shows that skin volatiles show a strong predictive ability for age (explained variance of 68%), stronger than other biochemical measures collected in this study (skin surface pH, water content) which are understood to vary with chronological age. Overall, this work provides new insights into the impact of aging on the skin volatile profiles which comprises both endogenously and exogenously derived volatile compounds. It goes toward demonstrating the biological significance of skin volatiles and will help pave the way for more rigorous consideration of the healthy “baseline” skin volatile profile in volatilomics-based health diagnostics development going forward.

## 1 Introduction

For years, biochemical, structural, and physical changes in aged-skin have been noted. In general, aging of human skin is driven by two processes: intrinsic processes, related to chronological aging, which occurs due to inherent genetics; and extrinsic processes, attributed to environmental and lifestyle factors, including UV exposure and diet.^1−4^ Oxidative damage, caused by an increased production of reactive oxygen species (ROS) in the skin, is one of the general mechanisms through which skin aging occurs.^5−8^ Age-associated exogeneous changes in the skin microbiome have been noted^9,10^ where increased bacterial diversity across skin sites with increasing age has been observed.^11,12^ Abundances of specific genera of bacteria such as *Lactobacillus*, *Cutibacterium*, and *Corynebacterium* have been shown to be altered with chronological age.^11,13^ Other well-established physiological changes in aged-skin include an increased skin surface pH^14−18^ and decreased water content and rates of transepidermal water loss (TEWL).^15^

Analysis of volatile compounds emanating from skin is emerging as an interesting source of information regarding subcutaneous and even systemic biochemistry.^19^ Volatile emissions from human skin originate from gland secretions and their interaction with microorganisms in the skin microbiome.^20^ This volatile emission is thought to contain over 600 compounds and comprises compound classes including alkanes, alkenes, aldehydes, acids, ketones and alcohols^21^ where individual compounds can reflect end-stage metabolic pathways and so can provide information on endogenous metabolic processes as well as potentially the microbial composition of the skin.^19^ Workflows used in the sampling and analysis of skin volatiles include our published method of headspace sampling using solid-phase microextraction (SPME),^22,23^ and other approaches such as the use of contact sampling using cotton/gauze pads and polydimethylsiloxane (PDMS) materials^24,25^ together with gas chromatography–mass spectrometry (GC-MS)^22,23,26^ as well as real-time MS techniques.^27^ Recently, however, HS-SPME sampling coupled with GC-MS analysis has emerged as a frequently used technique as it allows extraction and preconcentration in a single step in contrast to other sampling methods.

Some research publications to date have noted changes in the volatile profile related to participant age, specifically in the aldehyde emissions.^24,25^ Haze et al.^28^ identified the unsaturated aldehyde 2-nonenal, recovered from both male and female participants over 40, as a characteristic biomarker of aging. However, another subsequent study detected 2-nonenal in similar abundances across younger and older participants with no correlation being observed with age, weakening the case for it as a marker of age.^25^ Gallagher et al.^29^ observed significantly increased abundances of a different saturated aldehyde nonanal in older participants (over 40) compared to younger participants (under 40), where no significant differences were observed between male and female participants. In general, the volatile aldehyde emission from the body is considered a marker of oxidative stress^26^ resulting from an increased production of ROS. Fatty acids (FAs) such as palmitoleic, oleic, and vaccenic acid, present in sebum, have been shown to be increased in aged-skin.^30,31^ ROS induce peroxidation of these FAs to produce aldehydes, and thus, it can be hypothesized that increased aldehyde emissions from aged skin are linked to higher FA content and increased oxidative stress. Interestingly, aldehydes including nonanal and others such as decanal, benzaldehyde and 4-hydroxy-2-nonenal^32,33^ have been demonstrated to be activators of the Nrf-2-Keap1 pathway,^8,34^ a principle protective response to oxidative and electrophilic stressors, in human keratinocytes.^33^ Recovered abundances of other skin volatile compounds including sulfur-based compounds benzothiazole and dimethylsulfone and cosmetically derived compounds, such as hexyl salicylate and α-hexyl cinnamaldehyde, have also been shown to change significantly with age with increased abundances being recovered from younger participants. This may reflect increased cosmetic product usage in younger people.^29^

Machine learning (ML) and multivariate statistical approaches are now routinely employed for the analysis of human volatilomics to include skin as well as breath emissions.^35−39^ Both unsupervised, such as principal component analysis (PCA)^37,40^ and hierarchical clustering (HCA)^41^ which have discriminatory power, and supervised learning, such as multiple linear regression (MLR), partial least-squares-discriminant analysis (PLS-DA),^42^ linear discriminant analysis (LDA),^42^ others,^35,36,40^ which allow classification have been frequently used. These approaches allow for analysis through reducing dimensionality and aid in discrimination, clustering, classification and correlation of VOCs that may be linked to disease^35,36,43^ and other physiological factors.^38,42^

To our knowledge, research on age-associated changes in the skin volatile profile is limited,^28,29^ and shows a lack of agreement on age-dependent volatiles. This work aims to build on earlier research by employing a larger healthy participant cohort than any study before to more comprehensively characterize the volatile emissions that show significance with age using a HS-SPME GC-MS workflow. The first aim of this work was to collect and profile the volatiles of each of the 60 participants of varying ages. Gender-associated differences in younger and older participants were investigated, and correlations between the selected compounds and age were assessed to identify significant compound-specific correlations with participant age. The ability of the age-significant compounds identified to predict age was investigated, and the predictive ability was compared to other biochemical parameters, skin surface pH and water content as measured by the tissue dielectric constant (TDC). Predicting age with good certainty via the skin volatile profile will go toward demonstrating the biological significance of skin volatiles and allows us to understand baseline volatile profiles as a function of age. Gaining a clear picture of this profile is critical before skin volatiles can confidently be exploited in health diagnostics and other applications in the future.

## 2 Materials and Methods

### 2.1 Participant Profile and Skin Volatile Emission Sampling

**Figure 1:**
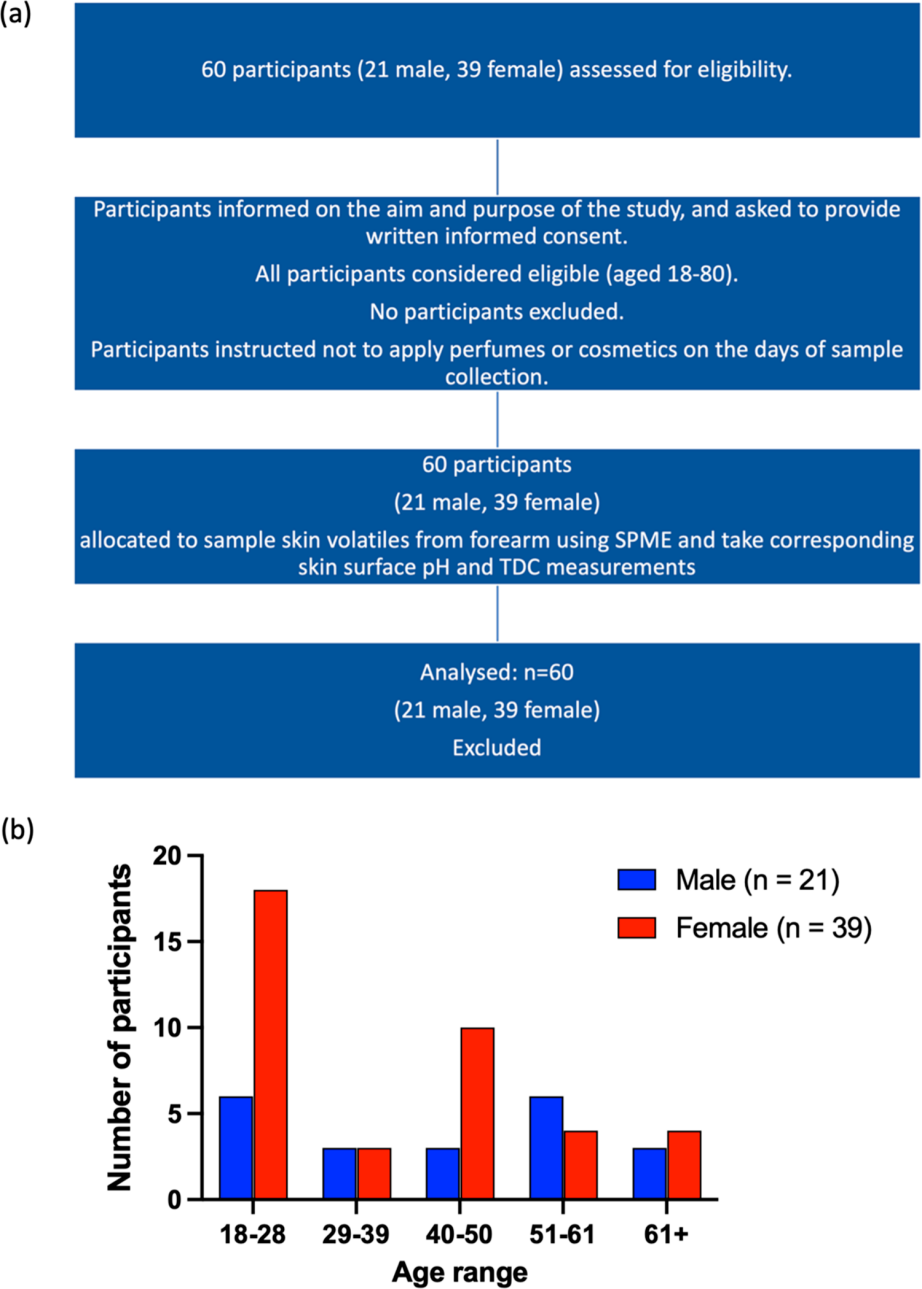
(a) Consort diagram for participant study and (b) number of male and female participant samples analyzed, categorized into age ranges of 10 years.

60 healthy volunteers, aged 18–78 (39 female, 21 male), were recruited (Figure 1). No special dietary regimes were imposed, however, participants were asked not to apply perfumes or cosmetics on their arms on the day of sampling. Participants were informed on the aim and purpose of the study and asked to provide written informed consent and complete a short questionnaire about their gender, age and cosmetic/fragrance use. The local ethics committee (Dublin City University Research Ethics Committee) approved the study on skin volatiles prior to commencement of the work (DCUREC/2016/053), and the study was performed according to the Declaration of Helsinki. Solid-phase microextraction (SPME) fibers were used for sampling volatiles in a headspace (HS) above the skin using a method described previously.^22^ Briefly, SPME fibers comprised 50/30/20 μm divinylbenzene/carboxen/polydimethylsiloxane Stableflex (2 cm) assemblies (57348-U, Supelco Corp., Bellefonte, PA, USA). The SPME fiber was housed within a glass HS affixed to the volar forearm with Leukosilk surgical tape (BSN Medical GmbH, Hamburg, Germany). The HS comprised a glass funnel (3 cm^3^ volume, Pyrex, Fisher Scientific Ireland) and two septa (Supelco Thermogreen LB-2 Septa plug, Merck, Ireland) where the septa served to hold the exposed SPME fiber above the skin in the enclosed HS (SI Figure 1). SPME fibers were exposed to the skin for 15 min, after which the fiber was retracted and removed from the HS and transferred into the GC injector for desorption. A sampling time of 15 min was employed based on an optimization study reported previously.^22^ One sample from the left or right volar forearm was taken for each participant. All samples were collected between November 2021 and February 2022.

**Figure 2:**
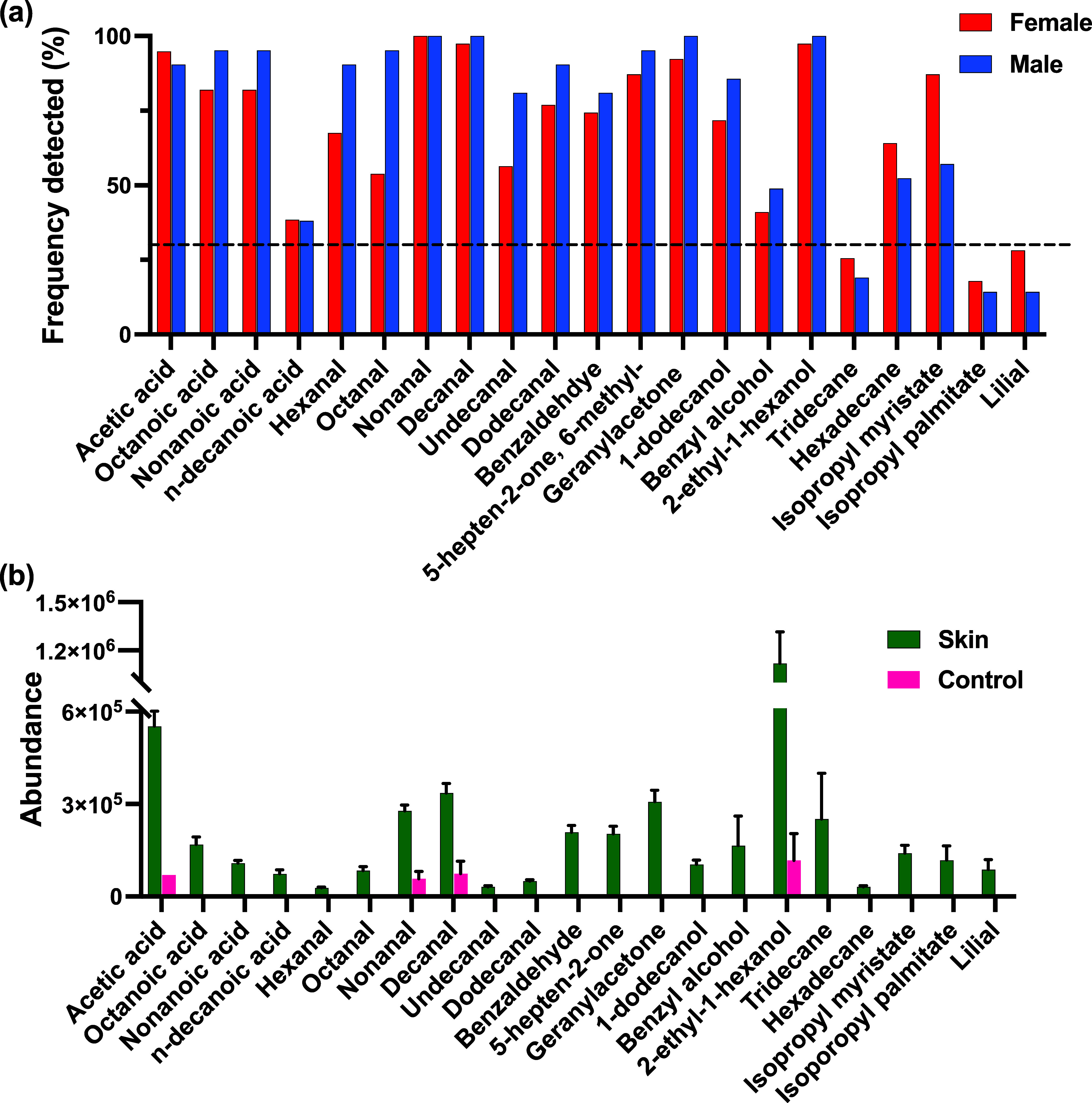
(a) Percent frequency of each VOC recovered from male (*n* = 21) and female (*n* = 39) participants and (b) bar chart illustrating the mean abundance of each compound recovered (*n* = 60 participants) compared to the blank HS (*n* = 6). Error bars represent the standard deviation in mean abundances recovered.

Blank air samples (in the absence of skin) were collected using the same glass HS used to sample skin. In this case, the glass HS was fully enclosed by wrapping in aluminum foil and parafilm and was sampled in the same manner as for skin (*n* = 6). Acetic acid, nonanal, decanal and 2-ethyl-1-hexanol were detected in low abundances that were not considered significant compared to abundances recovered from skin (Figure 2b).

### 2.2 Standard Calibration Curves

Specified concentrations of nonanal (CAS: 124-19-6, purity: 95%) and decanal (CAS: 112-31-2, purity: > 98%) standards (Merck, Ireland), were prepared individually in *n*-hexane and 1 μL volumes of these solutions were pipetted into separate 20 mL glass vials. Glass vials were sealed and the solutions were allowed to evaporate at 37 °C for 10 min. After 10 min, complete evaporation was assumed, and using the ideal gas law,^44^ the HS concentrations of both nonanal and decanal in 6 separate vials were recorded as 0.625, 1.25, 2.5, 5, 10, and 20 ppb. Finally, the HS of the vials were sampled using a SPME fiber for 15 min at 37 °C. After 15 min, the SPME fiber was retracted, removed and transferred to the GC injector for desorption. All standard preparations and analyses were performed in triplicate.

### 2.3 Gas Chromatography–Mass Spectrometry Analysis

An Agilent 7820A gas chromatograph connected to an Agilent 5977B mass selective detector (Agilent Technologies, Inc., Santa Clara, CA, USA) was used for all analyses. Separations were performed on an SLB-5 ms column (30 m × 0.25 mm × 0.25 μm df; Supelco). Helium carrier gas was used throughout this work, with a constant flow rate of 1 mL min^–1^. The system was equipped with a SPME Merlin Microseal (Merlin Instrument Company, Newark, DE, USA), and the inlet was maintained at a temperature of 250 °C. Splitless injection was used for all samples, and each SPME fiber was desorbed for 2 min within a SPME inlet liner (Supelco). The initial GC oven temperature was 40 °C for 5 min after which the oven was temperature-programmed to increase at a rate of 10 °C min^–1^ to 270 °C. The MS was operated at a scan rate of 3.94 s^–1^, with a scan range of 35–400 *m*/*z*, ion source temperature 230 °C and ionizing energy of 70 eV.

### 2.4 Data Analysis

Agilent MassHunter Qualitative Analysis 10.0 software was used to analyze raw chromatographic data. Peak acquisition and the respective peak area data were calculated by employing the chromatogram deconvolution compound mining algorithm. A peak filter of ≥10 000 counts was set. A Level 2 putative identification of compounds and structures was performed using the National Institute of Standards and Technology (NIST) library, supported by a visual comparison of the unknown mass spectra with previous literature reports and with retention index (RI) matching with a tolerance of ±15 RI units. A standard mixture of saturated alkanes (C_7_–C_30_), (Merck, Ireland) was used for RI matching. In addition to this, confirmation of the retention time (RT) of compounds including those that were shown to be significant with respect to age was done using commercially available standards (acetic acid (CAS: 64-19-7, purity: 99%), octanoic acid (CAS: 124-07-2, purity: 99%), nonanoic acid (CAS: 112-05-0, purity: 97%), hexanal (CAS: 66-25-1, purity: 98%), octanal (CAS: 124-13-0, purity: 99%), nonanal (CAS: 124-19-6, purity: 95%), decanal (CAS: 112-31-2, purity: >98%), benzaldehyde (CAS: 100-52-7, purity: >99.5%), 5-hepten-2-one, 6-methyl- (CAS: 110-93-0, purity: >99%), geranylacetone (CAS: 689-67-8, purity: > 97%) and 2-ethyl-1-hexanol (CAS: 104-76-7, purity: >98%), benzyl alcohol (CAS: 100-51-6, purity: >99.8%) and undecanal (CAS: 112-44-7, purity: >98%) (Merck, Ireland). Furthermore, compounds deemed to be contaminants (e.g., siloxanes likely arising from SPME fibers and column bleed) were excluded from data sets.

RStudio (version 2023.03.0) and Prism (version 9.4.0) were used for all data exploration and visualization. A Shapiro-Wilk’s test was first carried out in order to determine if the data had a normal or non-normal distribution using the R package “ggpubr”. This test confirmed the data set has a non-normal distribution, thus nonparametric techniques were employed for data exploration. Wilcoxon testing was used to determine significant difference and p-values <0.1 were deemed statistically significant. Spearman correlation analysis was carried out using R package “corrplot” (version: 0.92). Canonical correlation analysis (CCA) was carried out using “cca” (version: 1.2.1) and “ccp” (version: 1.2). Multiple linear regression (MLR) was carried out on data using “MASS” (version 7.3). Other R packages used included: “tidyverse”, “ggplot2”, and “ggfortify”.

### 2.5 Skin Surface pH and Tissue Dielectric Constant Measurements

Following sampling of volatiles, skin surface pH and tissue dielectric constant (TDC) measurements were collected at the same skin site. All measurements were carried out in triplicate. Skin surface pH measurements were collected by using a wireless HALO flat glass probe (HI14142) (Hanna Instruments). TDC measurements at an effective measuring depth of 0.5 mm were collected capacitively using a Delfin MoistureMeter D probe (Delfin Technologies, Kuopio, Finland).

## 3 Results and Discussion

### 3.1 Characterizing the Skin Volatile Profile in a Healthy Participant Cohort

Skin volatile emissions from the volar forearm of 60 participants were analyzed in this study. Consistent with previous studies,^22,23,26^ a variety of compound classes including acids, aldehydes, ketones, alcohols, hydrocarbons and esters were recovered. In agreement with earlier studies,^45,46^ high variability in the composition of the skin volatile profile across participants was observed, highlighting variance in interparticipant sampling as well as potential dependent variables such as age and gender that are investigated here as impacting abundances across the data set (SI Figure 2).

Twenty-one compounds were reliably identified across the samples, identified using a combination of RI matching and analytical standards (SI Table 1). Figure 2a shows the % frequency detected for each compound across participants. Among these are frequently reported skin volatile compounds acetic acid, octanoic acid, nonanoic acid, geranylacetone, 6-methyl-5-hepten-2-one, nonanal and decanal,^22,47−49^, all detected in >80% participants. Acetic acid, a short chain fatty acid (SCFA) is a primary microbial metabolite with many studies reporting it as a feature of the skin volatile profile.^21,49,50^ It is produced via the catabolism of skin lipids into long-chain FAs which are then further broken down by bacteria including *Propionibacteria* and *Staphylococci* that are present on skin.^51^ Medium chain FAs including octanoic-, nonanoic- and n-decanoic- acids were also recovered within this study and are well-established as components of sebum, produced by sebaceous glands.^31,52^ Six saturated- and 1 aromatic- aldehyde were recovered frequently across male and female participants. Benzaldehyde was recovered in approximately 75% of male and female participants. Its production within skin has been linked to benzyl alcohol oxidation and is considered as a microbial metabolite.^53−55^ As discussed earlier, aldehyde emissions from skin are considered important as end-products of lipid peroxidation reactions initiated by oxidative stress.^56^ Given their significance, and as nonanal and decanal were the most frequently recovered compounds here (97–100%), their emission fluxes were estimated using standard calibration curves (>SI Figure 3) as outlined in the Methods Section. Emission fluxes were calculated based on the sampling time and area of skin sampled and are reported in fmol cm^–2^ min^–1^. The calculated emission flux range across participants was 105–1130 fmol cm^–2^ min^–1^ for nonanal and 85–1333 fmol cm^–2^ min^–1^ for decanal. The lower end of the flux range is in broad agreement with fluxes previously reported^26,57,58^ and, as discussed later on, the lower end of this range was most strongly associated with younger participants. Upper ranges of fluxes measured here are higher than what has been published to date, potentially linked to the fact that other studies may have recruited younger participants predominantly; however, little detail is available on age distributions of participants recruited for these earlier studies. It is also possible that the fluxes measured here may be overestimated due to loss of analyte to the HS glass surface during calibration point measurements. This would result in lower than expected recovered abundances potentially leading to some overestimation of the emission fluxes.

Ketones recovered included 6-methyl-5-hepten-2-one, and geranylacetone, which are among the most frequently reported skin volatiles,^21^ and recovered here from almost 100% of participants. As outlined above, no dietary restrictions were imposed on this study as there is no known influence of diet on the compounds that we have selected. Other compounds such as acetone^59^ and some sulfur compounds,^24^ none of which were detected in this study have been shown to be linked to diet control/changes. 2-ethyl-1-hexanol was also recovered in almost 100% of participants with high abundances (Figure 2b) and has been previously reported as a microbial degradation product of plasticizers present in indoor air and cosmetics.^60,61^ Other compounds believed to be from exogenous sources include isopropyl myristate, isopropyl palmitate and lilial^29^ were also recovered at high frequency rates. A requirement for further consideration in this study was a frequency of detection threshold of >30%. This excluded tridecane, isopropyl palmitate and lilial for both genders. The final data set used for all subsequent data analysis consisted of 18 frequently recovered compounds.

### 3.2 Gender Influence on Skin Volatile Profile across Young and Old Participants

**Figure 3:**
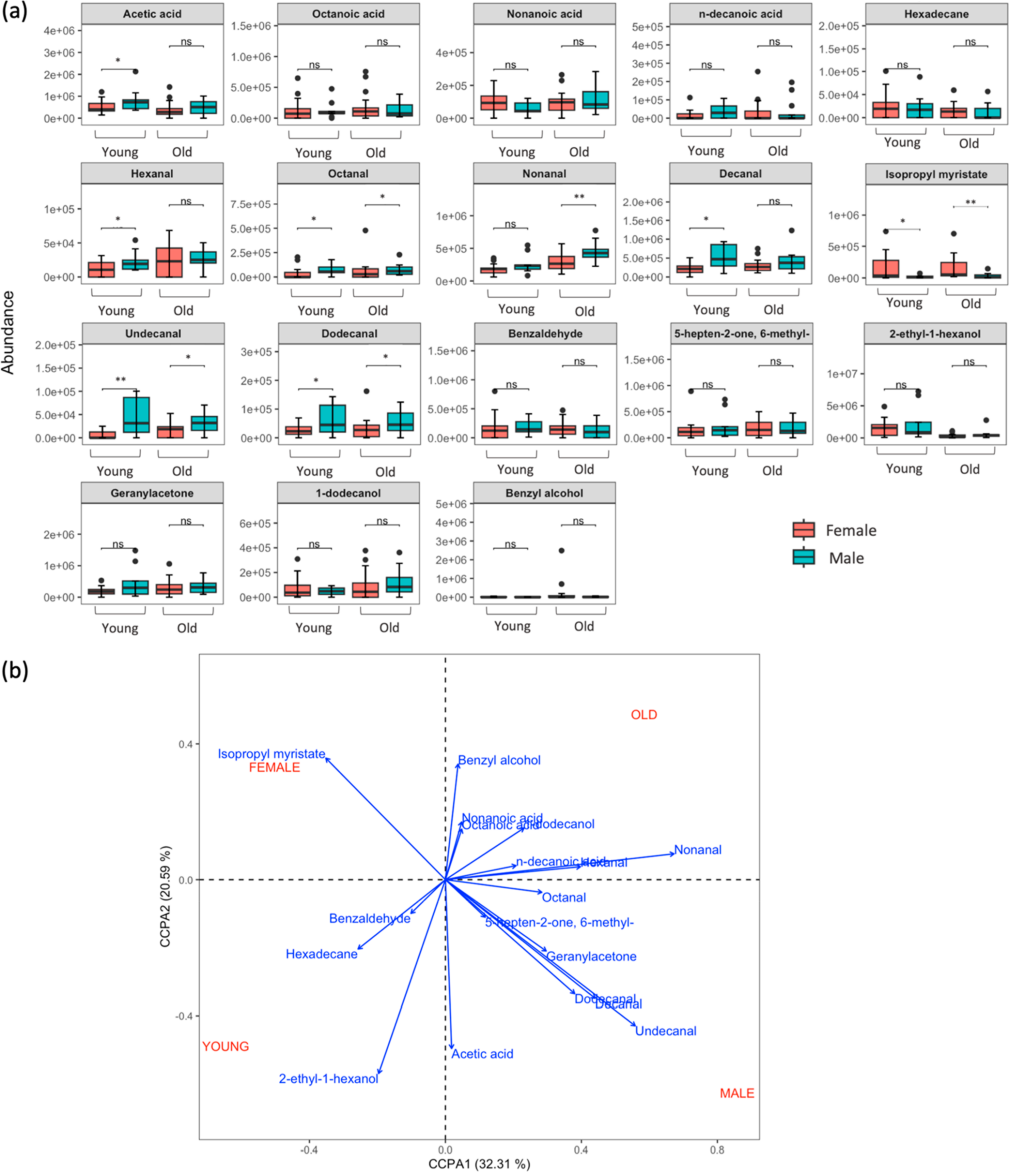
(a) Grouped boxplots comparing the abundances of the 18 selected compounds emitted by males (*n* = 9) and females (*n* = 21) aged 18–40 (young); and males (*n* = 12) and females (*n* = 18) aged 40–80 (old). *Y*-axis labels are displayed in scientific notation where *ae* + *b* = *a* × 10^*b*^. Statistical significance was calculated using the Wilcoxon signed rank test (ns: *p* > 0.1; *: *p* ≤ 0.1; **: *p* ≤ 0.01). Error bars represent standard deviation in recovered abundances and (b) CCPA ordination of the 18 selected compounds in relation to categorical variables describing age brackets (young is defined as <40, old is defined as >40) and gender (*n* = 60 participants; male *n* = 21, female *n* = 39).

Studies have shown that the human skin volatilome is influenced by gender.^23,42^ Our group,^23^ along with others^62^ have shown a differences in recovered abundances of medium and long-chain FAs between males and females. Increased abundances of aldehydes and ketones in male participants have also been reported compared to females.^23^ In order to investigate if gender had an influence on the recovered volatiles across age, male and female participants were categorized into groups according to age range; young (aged 18–40) and old (aged 40–80), similar to Gallagher et al.’s study.^29^ Comparative boxplots (Figure 3a) showing compound-level data were constructed and significant differences (*p*-value < 0.1) in emissions between young males and females and old males and females were investigated. Significantly higher recovered abundances of octanal, undecanal and dodecanal can be observed in males compared to females, irrespective of being young or old. Other aldehydes such as hexanal and decanal were recovered in significantly higher abundances in younger males compared to younger females but were not significant across gender for older participants. Nonanal was recovered in higher abundances in older males compared to older females but no significant gender difference was noted for younger participants. Acetic acid was also recovered in a significantly higher abundance in young males compared to young females. It was not significant for gender in older participants. Acetic acid abundances were also investigated for their correlation with the skin surface pH and young males were noted to have the lowest pH of all cohorts (SI Figure 4) which correlates with the highest acetic acid abundances. Younger and older females were observed to have greater recovered abundances of isopropyl myristate compared to younger and older males, which could be due to the presence of this compound as an emollient in cosmetic products being used more frequently by the female participants.^63^

Supporting these findings, canonical correspondence analysis (CCPA) was used to model the relationship between recovered volatile compound abundances (as quantitative variables) (*n* = 18) and a defined set of categorical variables (male, female, young, old). Figure 3b shows the CCPA plot and demonstrates where maximum correlation occurs, highlighting which volatile compounds are most associated with being male, being female, and being young and being old, by means of the quadrant direction and length of each arrow. Results of this analysis further support the findings in Figure 3a where higher abundances of decanal, undecanal, and dodecanal correlate most strongly with being male, while isopropyl myristate is most correlated with being female (irrespective of age). In terms of the correlation of volatile variables with being young and being old, higher 2-ethyl-1-hexanol abundances was shown to correlate most strongly with younger age groups. A high acetic acid abundance was noted to correlate with being a young male while a high nonanal abundance correlated most with being an older male.

To summarize, gender as well as being young/old was shown to influence skin volatile emissions. The data suggests that volatile profiles of males and females and old and young participants differed in terms of abundances of specific compounds including acetic acid, hexanal, octanal, nonanal, decanal, undecanal, dodecanal, and isopropyl myristate. Therefore, for subsequent work, when investigating the effect of age on volatile emissions, we examined genders independently.

### 3.3 Skin Volatile Compound Significance for Age and Known Age-Dependent Physiological Parameters

**Figure 4:**
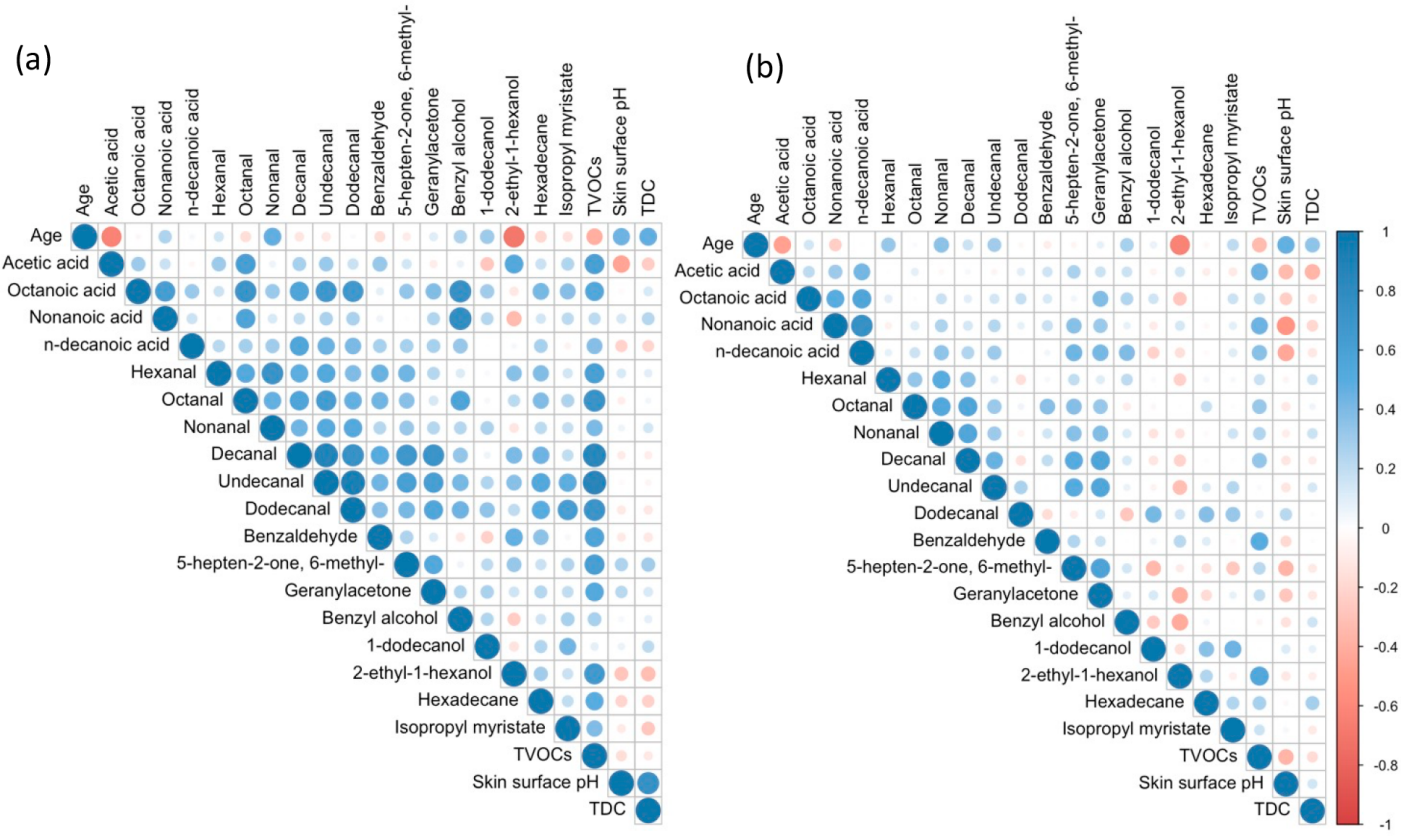
Spearman correlation plot for age, individual VOCs, TVOCs, skin surface pH and TDC for (a) male (*n* = 21) and (b) female (*n* = 39) participants. Blue color indicates a positive correlation, and red color indicates a negative correlation according to scale bar. Circle diameter represents the magnitude of the correlation.

Correlations between age, skin surface pH, TDC, and all 18 volatiles (Section 3.1) were investigated (Figure 4) and results are summarized in Table 1 and SI Table 1. In terms of volatile compound correlation with age, the highest correlation coefficient magnitude was seen for 2-ethyl-1-hexanol (*r* = 0.6–0.7), which is highly significant in this study across both genders. While this compound is not likely derived from endogenous sources, it is known to be produced by microbial degradation of plasticizers such as diethyl phthalate (DEP), typically present in indoor air and also fragrances and cosmetic products.^64,65^ These plasticizers may be metabolized by skin bacteria to produce 2-ethyl-1-hexanol.^61^ However, to the best of our knowledge, this process has yet to be demonstrated for any skin commensal. The skin microbiome undergoes significant shifts over time, resulting in shifts in skin’s metabolic activity. This could potentially be associated with decreased 2-ethyl-1-hexanol emissions in older participants.^66^ Furthermore, 2-ethyl-1-hexanol itself has been reported as a component of fragrances and so it also could be speculated that its higher abundances in younger participants may be linked to a more frequent use of fragrances compared to older participants.^67^ However, in contrast to this, other fragrance-derived volatile compounds, such as isopropyl myristate, showed no significance for age. Further work into the elucidation of the source of 2-ethyl-1-hexanol within skin could be carried out by conducting in vitro experiments to confirm the microbial production of 2-ethyl-1-hexanol by culturing commensal organisms in ^13^C-labeled media and assessing if ^13^C is subsequently present in the 2-ethyl-1-hexanol detected in the HS.

**Table 1:** Correlation Coefficients and *p*-Values for Male and Female Participants for 18 Volatiles, TVOCs, Skin Surface pH, and TDC as Functions of Age^a^

	Correlation (coefficient *r*, p-value)	
	Age	
Variable	Male	Female
**Acetic acid**	**-0.622, 0.002↓**	**-0.469, 0.002↓**
Octanoic acid	–0.031, 0.895	0.146, 0.375
Nonanoic acid	0.259, 0.256	–0.237, 0.146
*n*-decanoic acid	0.033, 0.885	0.006, 0.969
**Hexanal**	0.145, 0.530	**0.320, 0.046**
Octanal	–0.166, 0.472	0.024, 0.888
**Nonanal**	**0.473, 0.030↑**	**0.359, 0.024↑**
Decanal	–0.124, 0.591	0.166, 0.313
**Undecanal**	–0.118, 0.611	**0.290, 0.073↑**
Dodecanal	0.028, 0.904	–0.031, 0.852
Benzaldehyde	–0.165, 0.475	–0.081, 0.622
6-Methyl-5-hepten-2-one	–0.094, 0.684	–0.042, 0.800
Geranylacetone	0.107, 0.645	0.074, 0.653
**Benzyl alcohol**	0.259, 0.256	**0.279, 0.085↑**
1-Dodecanol	0.307, 0.176	0.070, 0.673
**2-Ethyl-1-hexanol**	**-0.692, 0.0005↓**	**-0.621, 0.00002↓**
Hexadecane	–0.196, 0.393	–0.039, 0.815
Isopropyl myristate	–0.143, 0.536	0.206, 0.208
**TVOCs**	**0.401, 0.029↑**	**-0.333, 0.033↓**
**Skin surface pH**	**0.440, 0.045↑**	**0.465, 0.002↑**
**TDC**	**0.476, 0.029↑**	**0.342, 0.033↑**

a Variables which show significant change (*p* < 0.1) with age are in bold; arrows represent up-regulation (↑) or down-regulation (↓).

A decrease in the recovered acetic acid abundance was observed with increasing age for both genders. As discussed earlier, acetic acid is a microbial breakdown product of long-chain FAs and specific bacteria associated with the breakdown of acetic acid have been shown to decrease in abundance with aging;^11,13,68^ this may be linked to the decrease in acetic acid emission with age observed here. No significant age effects were noted for the other acids. Interestingly, skin surface pH significantly increased with age for both males (skin surface pH: *p* = 0.045) and females (skin surface pH: *p* = 0.002), which agrees with earlier literature^18,69^ and is consistent with the observed decrease in acetic acid. This pH decrease with age been linked with various mechanisms^70,71^ which are responsible for the acidification of skin, and which are thought to be disturbed in older skin.^14−16,18,70,72^ As discussed above, the skin surface pH has been shown to be modulated by specific acids emitted from the skin. While there is an obvious correlation between skin surface pH and acetic acid abundance for both males (*p* = 0.044) and females (*p* = 0.038), it is also interesting to note that some longer-chain acids such as such as nonanoic (females; p = 0.0006) and n-decanoic acid (females; *p* = 0.006) were shown to correlate with skin surface pH in female participants only (Figure 4, SI Table 2). This may indicate that acetic acid is likely the acid dominating skin surface pH modulation, and this may be linked to its increased expression in skin relative to the longer-chain acids (Figure 2b).

Nonanal, a lipid peroxidation product of oleic acid, was observed to be significantly up-regulated with increasing age in both males and females. This is likely linked to increased oxidative stress in skin associated with aging.^29^ Hexanal and undecanal were also up-regulated in older females (*p* = 0.046, *p* = 0.073, respectively) but not in older males. Hexanal, again, is a lipid peroxidation end-product arising from the breakdown of linoleic and palmitoleic acids, triggered by an increase in ROS. Undecanal is an end-product of lipid peroxidation of *cis*-heptadec-6-enoic acid, which is less abundant in sebum compared to oleic and palmitoleic acids,^73^ potentially explaining its lower abundances recovered here relative to the other aldehydes.

Benzyl alcohol, up-regulated in older females in this study, is a frequently reported skin emission^25,45,49^ which has been detected in the HS of cultured *Staphylococcus epidermidis*,^49^ indicating its potential as being a skin microbial metabolite. Furthermore, benzyl alcohol can be oxidized by microbes to produce benzaldehyde.^53,55^ It could be speculated that alterations in the skin microbiome with age may influence this process; however, no significant abundance changes in benzaldehyde were noted with age in this study. Finally, no significant change in abundance with age for either gender was observed for ketones, hydrocarbons, or esters recovered.

TDC is also known to increase with age, related to changes in the skin where there is a shift in water state governed by altered protein folding, that allows more free water to be present in older skin.^74−76^ It was hypothesized that the TDC value measured may influence the partitioning of compounds between the aqueous compartment of skin and the HS above skin. However, our data show no correlation between TDC and total volatile organic compounds (TVOCs) for either males and females. More polar compounds, e.g., acetic acid, benzyl alcohol and 2-ethyl-1-hexanol, were investigated individually for their correlations with TDC. Given their more hydrophilic character and higher octanol–water coefficients (*K*_ow_), it was hypothesized that these molecules would partition more strongly into the free water in the skin, reflected in a decreased volatile emission flux. However, no correlation was observed. One reason for this may be related to the volume of water available from skin being significantly smaller than the volume of the HS (3 cm^3^) so that any variation in skin water content impacting VOC partitioning changes may not be significant enough relative to the HS volume to be observed in this study.

In summary, recovered abundances of 6 volatile compounds (acetic acid, hexanal, nonanal, undecanal, benzyl alcohol, and 2-ethyl-1-hexanol), as well as skin surface pH and TDC (as expected) were shown to vary significantly with participant age in males and/or females.

**Figure 5:**
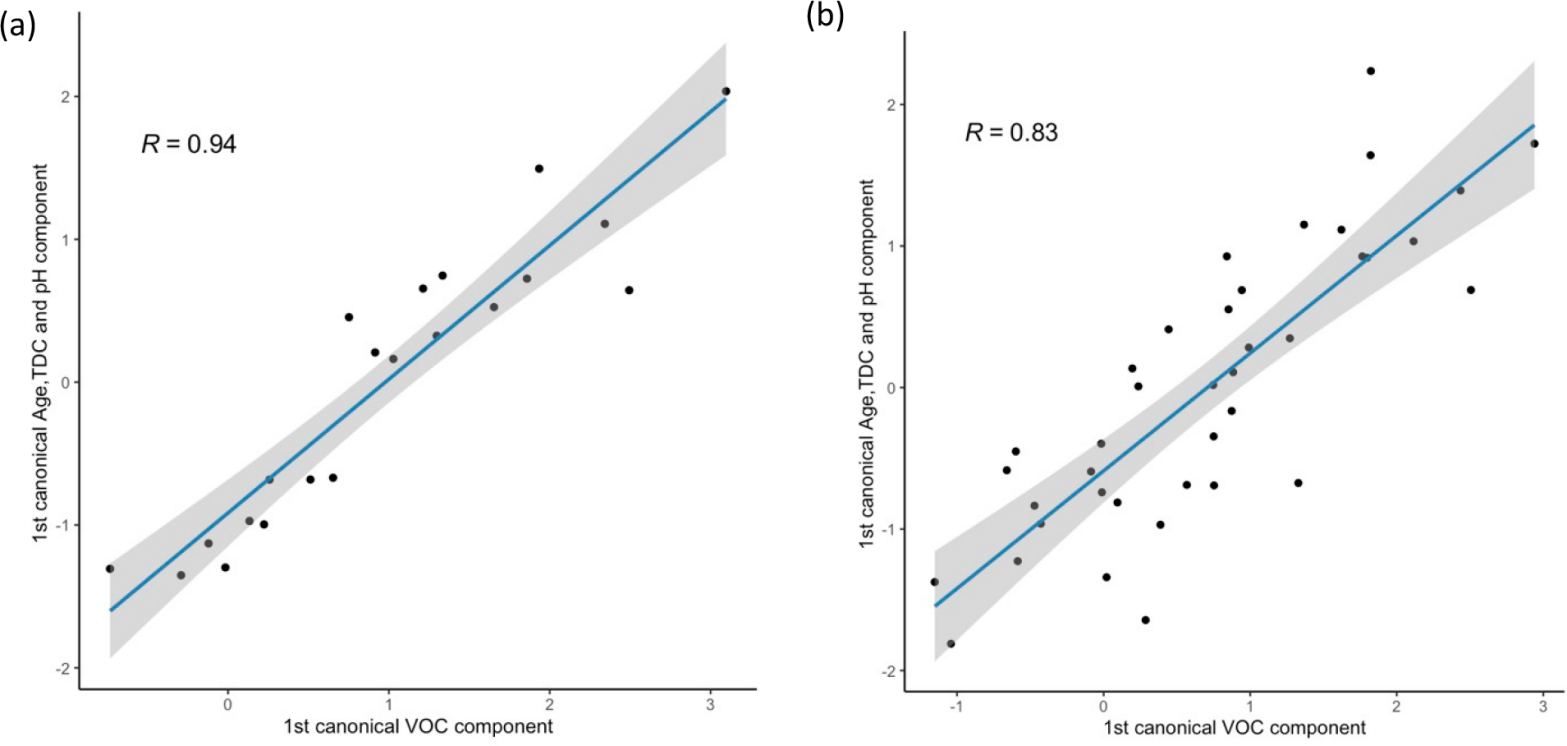
CCA score plots for parameters age, skin surface pH, and TDC (*y*-axis) and the selected VOCs (*x*-axis) using the first canonical components for (a) male (*n* = 21) and (b) female (*n* = 39) participants. Gray area shows the 95% confidence intervals of the CCA scores plots.

CCA, an extension of the bivariate Spearman correlation analysis, was used to quantitatively assess the maximum correlation between a combined linear variable component (age, TDC and pH) and the VOC variable component (Figure 5). The VOC variable component (*x*-axis) is composed of the 6 identified age-significant volatiles (Table 1). Figure 5 shows the CCA scores plots for males and females, where each data point represents the correlation of the combined data of age, skin surface pH, and TDC with the volatile profile of a single participant. Correlations of 0.936 between the canonical variates for male participants and 0.831 between the components for female participants were found. The lower correlation between components observed for female participants is at least in part due to the high variability in the younger female profiles.

**Figure 6:**
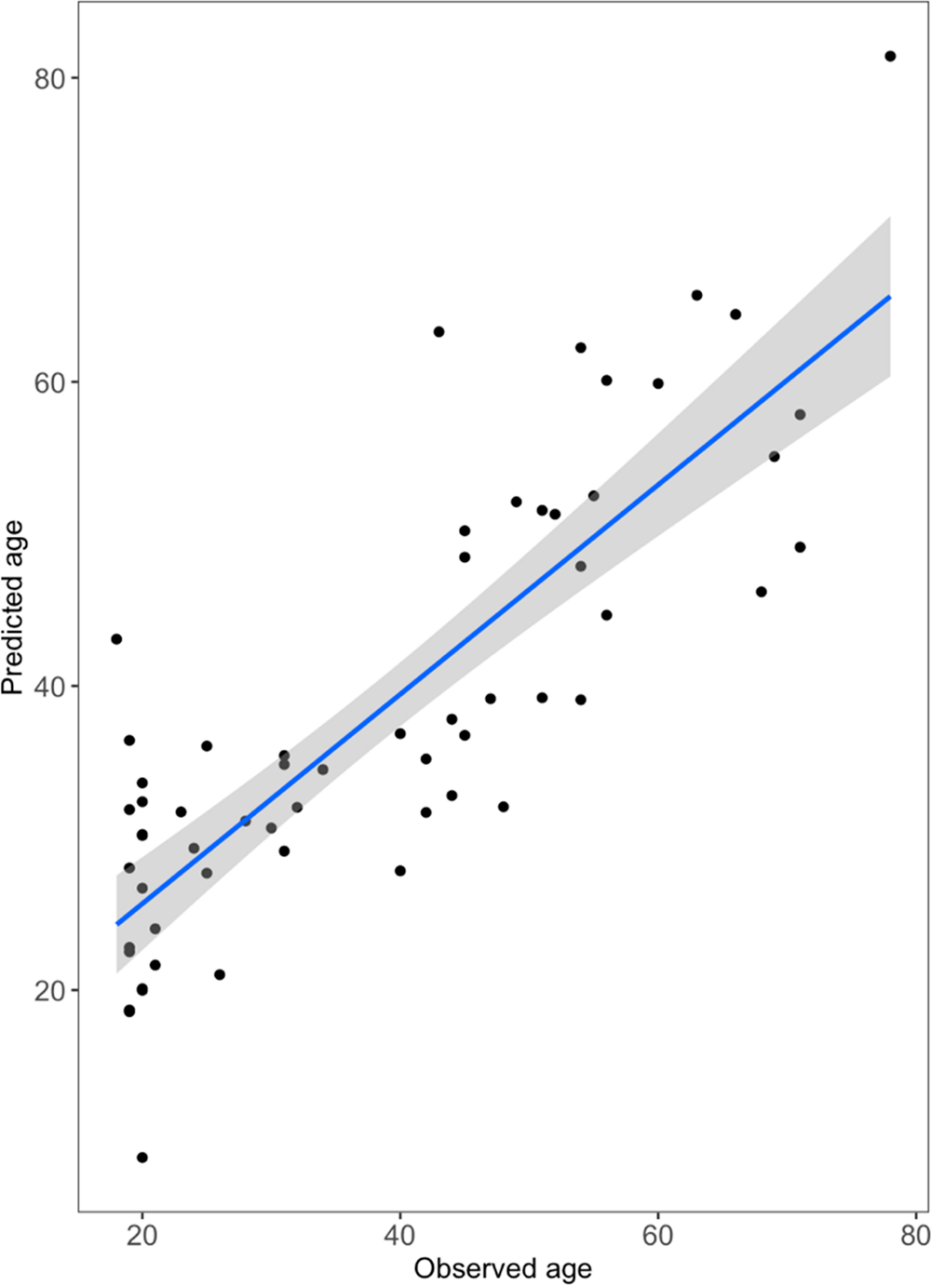
MLR predictive model for age using age-significant VOCs and gender only as independent variables (see Table 2) for *n* = 60 participants (male; *n* = 21, female; *n* = 39). Gray area shows the 95% confidence intervals of the model.

Finally, multiple linear regression (MLR), a statistical approach that uses several variables to predict the outcome of a response variable, was employed to determine how well participant age could be predicted based on the data collected in this study. First, an age-regression model with pH and TDC as parameters already established to change with age and participant gender was constructed. 28% of the variance in the data was explained using these variables (Table 2). To investigate if VOC abundances could explain additional variance, a model (Table 2, Figure 6) comprising the 6 key volatiles identified from this study (Table 1) and participant gender as variables was built. This model accounted for a much greater amount of variance, 68% (Table 2). This illustrates the ability of skin VOCs to predict age as compared with that of these other physiological skin parameters already well-established to vary with age. Finally, all variables were included in the MLR model to maximize the ability to predict age based on the skin data collected. This showed a variance of 75%, an improvement beyond either the VOCs or the pH and TDC models alone. These models, even those based on these VOCs only, have the capability to predict chronological age of a participant with reasonable accuracy. This serves to highlight the importance of age, as well as gender, when defining the healthy skin VOC profile and seeking to understand deviations from this profile after interventions or in disease states.

**Table 2:** Variance Explained (%) and Approximate Error Rate (%) for the MLR Models Constructed^a^

Independent variables	% Variance explained (*R*^2^)	Approx. error rate (%)
Skin surface pH, TDC, gender	28	39
Skin VOCs (acetic acid, hexanal, nonanal, undecanal, benzyl alcohol, 2-ethyl-1-hexanol), gender	68	26
Skin VOCs (acetic acid, hexanal, nonanal, undecanal, benzyl alcohol, 2-ethyl-1-hexanol), skin surface pH, TDC, gender	75	24

a Regression equations given in SI Table 3.

## 4 Conclusion

In this work, comprehensive skin volatilomic data was obtained from a large healthy participant cohort using a HS-SPME GC-MS workflow with a total of 18 compounds, of both endogenous and exogenous sources, identified in the volatile emission from participants’ skin. Gender- and age-associated correlations were investigated using various multivariate analysis approaches, and results identified both gender- and age-influenced changes in the emission of specific volatile compounds, building on some earlier initial studies to highlight the significance of aging on the skin volatile profile. This work validates the earlier reporting of nonanal as a marker of age and uncovers the potential of a set of skin volatile compounds, upon which chronological age can be predicted. Indeed, the ability to predict participant age using volatiles was better than prediction using other skin barrier property measures (pH and TDC) that are known to be age-dependent. The 6 skin volatiles identified in this study exhibited greater predictive ability compared to these other biochemical parameters, thus demonstrating the biological significance of skin volatiles. Here, the MLR model describes variance within the age profile, providing a numerical solution based on 60 samples, where numbers of participants across the five age ranges defined were not controlled and hence not homogeneously populated. A larger and more balanced data set would help eliminate potential model bias/skewing due to such an issue. Furthermore, while the potential origin of each volatile metabolite is discussed here based on the existing literature, critical future work will involve in vitro investigations of specific mechanisms driving the production of these volatiles. Overall, however, this work gives us clearer insight into the age-dependent skin volatile signature. Such investigations are critical to progressing the application of skin volatiles in health biodiagnostics in the future.

## Supporting Information Available

Image of SPME fibre within the glass headspace (3 cm^3^) affixed to the volar forearm for skin volatile sampling; Distribution of compound classes recovered from each participant’s skin volatile samples based on relative chromatographic peak areas of the 21 identified compounds; Retention time, significant mass spectral peaks, NIST and calculated retention index (RI) values for each compound identified in the HS of skin samples (*n* = 60; age range: 18-78) after 15 min sample collection using the HS-SPME followed by thermal desorption to GC-MS; calibration curves for nonanal and decanal showing peak abundance vs mass (μg) present in the 3 cm^3^ glass headspace; grouped boxplots comparing skin surface pH and TDC between young females and young males, and old females and old males; results of Spearman correlation for male and female participants for acidic VOCs as a function of skin surface pH; MLR predictive model equations for *n* = 60 participants; gender is a categorical variable where the number 1 is input for male participants and 0 for female participants (PDF)
