# Supplementary material for: Predicting Chronological Age via the Skin Volatile Profile
Source: J Am Soc Mass Spectrom. 2024 Feb 7;35(3):421–32. doi: 10.1021/jasms.3c00315 (PMC10921460; doi:10.1021/jasms.3c00315)
Supplement: Supplementary file 1 — js3c00315_si_001.pdf [file js3c00315_si_001.pdf]

Supplementary Information

Predicting Chronological Age via the Skin Volatile Profile

Melissa Finnegan[1], Shane Fitzgerald[1], Romain Duroux[2], Joan Attia[2], Emma Markey[1], David O'Connor[1], Aoife Morrin[1]*

[1]School of Chemical Sciences, Insight SFI Research Centre for Data Analytics, National Centre for Sensor Research, Dublin City University, Ireland

[2]IFF-Lucas Meyer Cosmetics, Toulouse, Cedex 1, France

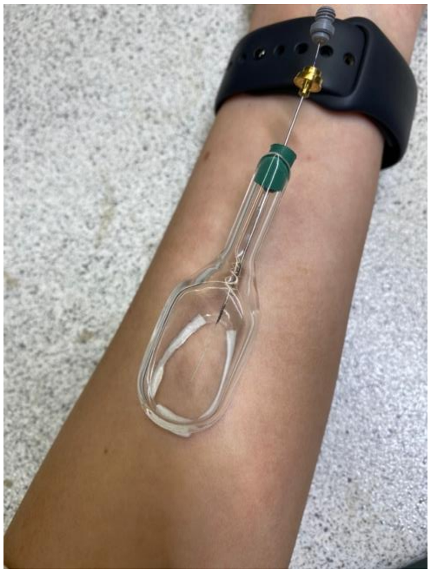

SI Figure 1. Image of SPME fibre within the glass headspace (3 cm^3) affixed to the volar forearm for skin volatile sampling (affixed with surgical tape which is not shown for the purposes of clarity).

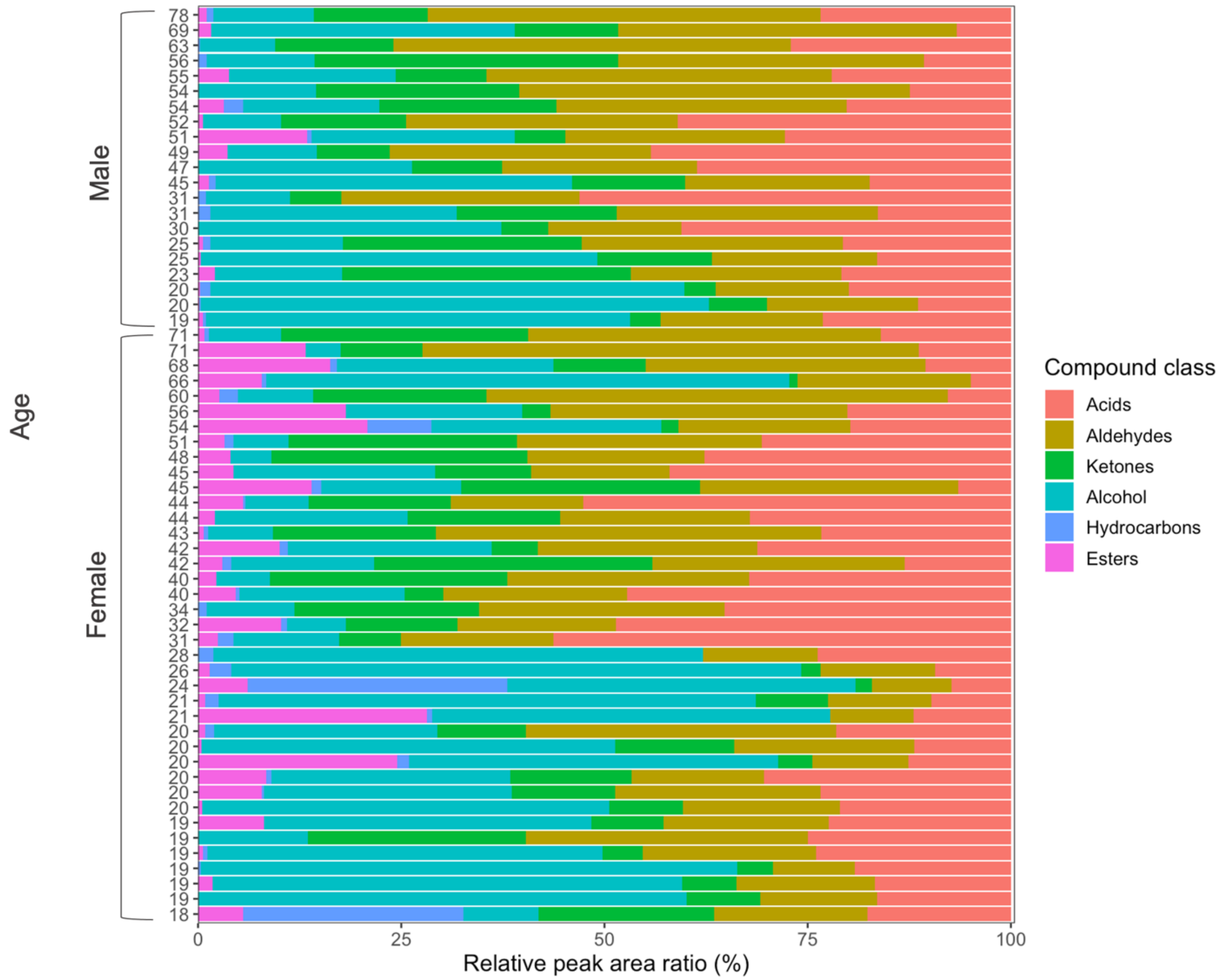

SI Figure 2. Distribution of compound classes recovered from each participant's skin volatile samples based on relative chromatographic peak areas of the 21 identified compounds.

SI Table 1. Retention time, significant mass spectral peaks, NIST and calculated retention index (RI) values for each compound identified in the HS of skin samples (n=60; age range: 18-78) after 15 min sample collection using the HS-SPME followed by thermal desorption to GC-MS. Compounds are listed in order of increasing retention time.

Compound	Chemical formula	Retention time (min)	MS spectral peaks (most abundant to least)	NIST RI	Calculated RI
Acetic acid	CH_3COOH	2.806	43,45,60	646	653
Hexanal	$C_6H_{12}O$	7.048	43,41,56	800	804
Benzaldehyde	C_7H_6O	11.128	106,77,51	957	966
6-methyl 5-hepten-2-one	$C_8H_{14}O$	11.496	43,108,69	991	986
Octanal	$C_8H_{16}O$	11.831	41,57,84	1005	1003
2-ethyl-1-hexanol	$C_8H_{18}O$	12.378	57,41,70	1026	1032
Benzyl alcohol	C_7H_8O	12.523	79,108,51	1034	1040
Nonanal	$C_9H_{18}O$	13.734	57,41,69	1105	1106
Octanoic acid	$C_8H_{16}O_2$	14.708	60,73,43	1175	1167
Decanal	$C_{10}H_{20}O$	15.369	57,41,70	1208	1208
Nonanoic acid	$C_9H_{18}O_2$	16.181	73,60,41	1272	1261
Tridecane	$C_{13}H_{28}$	16.733	57,43,71	-	-
Undecanal	$C_{11}H_{22}O$	16.853	41,57,69	1310	1308
n-decanoic acid	$C_{10}H_{20}O_2$	17.487	73,60,129	1362	1354
Dodecanal	$C_{12}H_{24}O$	18.236	43,57,69	1412	1409
Geranylacetone	$C_{13}H_{22}O$	18.689	43,69,41	1458	1457
1-dodecanol	$C_{12}H_{26}O$	19.072	55,43,69	1469	1472
Lilial	$C_{14}H_{20}O$	19.832	189,91,41	1532	1535
Hexadecane	$C_{16}H_{34}$	20.564	57,43,71	-	-
Isopropyl myristate	$C_{17}H_{34}O_2$	23.028	43,57,102	1824	1824
Isopropyl palmitate	$C_{19}H_{38}O_2$	25.027	43,102,57	2023	2015

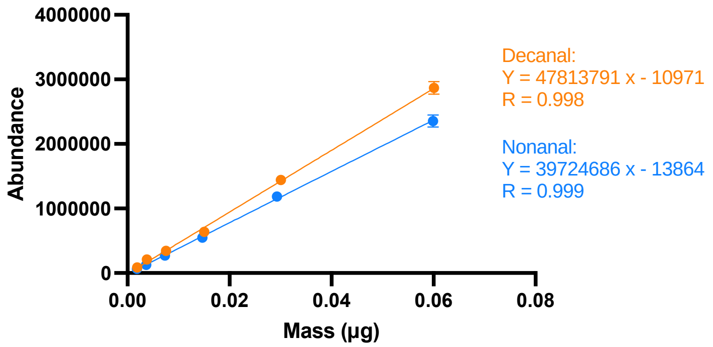

SI Figure 3. Calibration curves for nonanal and decanal showing peak abundance vs mass (µg) present in the 3 cm^3 glass headspace. Error bars represent standard deviation of n=3 replicates for each mass. Note: this data was used to calculate emission fluxes for nonanal and decanal.

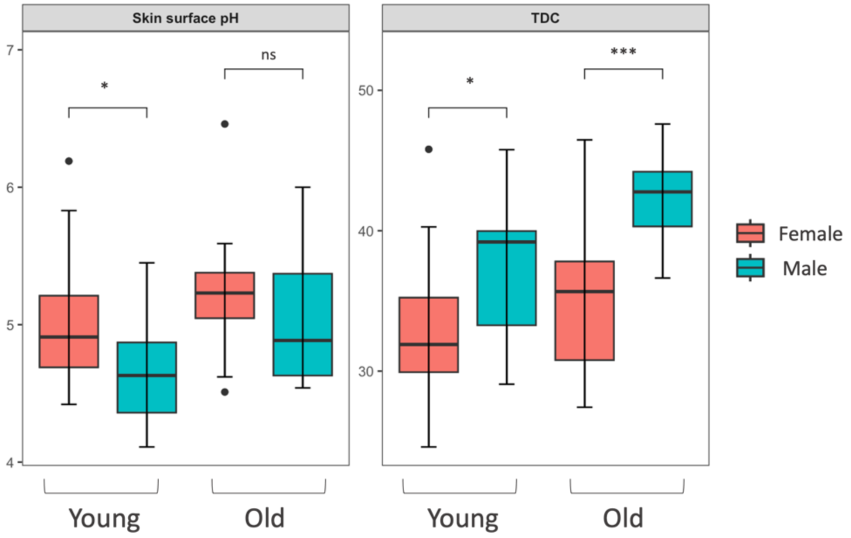

SI Figure 4. Grouped boxplots comparing skin surface pH and TDC between young females and young males, and old females and old males. Y-axis labels are displayed in scientific notation where ae+b = a × 10^b. Statistical significance was calculated using the Wilcoxon signed rank test (ns: $p > 0.1$; *: $p <= 0.1$; **: $p <= 0.01$, **: $p <= 0.001$). Error bars represent standard deviation in recovered abundances.

SI Table 2. Results of Spearman correlation for male and female participants for acidic VOCs as a function of skin surface pH. Variables which show significant change (p>0.1) with skin surface pH and TDC are bolded and arrows represent up-regulation (↑) or down-regulation (↓).

Variable	Correlation (coefficient *r*, p-value)	
	Skin surface pH	
	Male	Female
**Acetic acid**	**-0.443, 0.044 ↓**	**-0.333, 0.038↓**
Octanoic acid	-0.030, 0.898	-0.235, 0.1499
**Nonanoic acid**	0.136, 0.554	**-0.524, 0.0006↓**
**n-decanoic acid**	-0.215, 0.348	**-0.426, 0.006↓**

SI Table 3. MLR predictive model equations for n=60 participants. Gender is a categorical variable where the number 1 is input for male participants and 0 for female participants.

Independent variables	Regression equation
Skin surface pH, TDC, gender	$\text{Age} = -64.84 + 5.09\,(\text{gender}) + 14.48\,(\text{skin surface pH}) + 0.787\,(\text{TDC})$
Skin VOCs (acetic acid, hexanal, nonanal, undecanal, benzyl alcohol, 2-ethyl-1-hexanol), gender	$\text{Age} = 27.40 + 2.90\,(\text{gender}) - 7.62 \text{ x } 10^{-6}(\text{acetic acid}) + 6.96 \text{ x } 10^{-5}(\text{nonanal}) + 7.82 \text{ x } 10^{-5}(\text{hexanal}) - 5.02 \text{ x } 10^{-5}(\text{undecanal}) - 5.07 \text{ x } 10^{-6}\,(2 - \text{ethyl} - 1 - \text{hexanol}) + 1.25 \text{ x } 10^{-5}\,(\text{benzyl alcohol})$
Skin VOCs (acetic acid, hexanal, nonanal, undecanal, benzyl alcohol, 2-ethyl-1-hexanol), skin surface pH, TDC, gender	$\text{Age} = -33.58 + 3.18\,(\text{gender}) + 10.64\,(\text{skin surface pH}) + 0.141\,(\text{TDC}) - 1.99 \text{ x } 10^{-6}(\text{acetic acid}) + 6.93 \text{ x } 10^{-5}(\text{nonanal}) - 4.75 \text{ x } 10^{-5}(\text{hexanal}) - 2.39 \text{ x } 10^{-5}(\text{undecanal}) - 5.03 \text{ x } 10^{-6}\,(2 - \text{ethyl} - 1 - \text{hexanol}) + 1.30 \text{ x } 10^{-5}\,(\text{benzyl alcohol})$
